# Weakly Children

**Published:** 1883-11

**Authors:** 


### WEAKLY CHILDREN.

No subsequent care can fully atone for neglect of proper physical training and development in childhood. Some parents are so tender of their children that they hardly allow them to go out of their sight. They keep them in school nine months in the year, and under more or less restraint and confinement during the other three months. The result is that neither mind or body reach their full development ; and they are children at twenty, and weaklings the rest of their lives. The perfection of manhood is "a sound mind in a sound body." Most of the business of the great cities is in the hands of country-bred men, while those born and brought up in affluence in cities, are, as a rule, occupying inferior positions. This condition of things will not always remain thus ; parents are beginning to appreciate the cause and to seek the remedy. Early last Summer I went up among the hills of Sussex County, N. J., and stopped for a day or two with some friends who reside on a large farm a mile from the beautiful village of Newton. A delicate lad of ten years of age from Brooklyn had been sent to spend the summer with this family. When he came he had but little appetite, his eyes were weak and he could endure but little fatigue; but he entered into rural life with great gusto, and when he was sent for at the end of a month, he begged to remain longer. Another delicate boy about the same age was sent up from the city later in the season, and the two are full of business; racing over the hills, riding bare-back or in the farm wagons, and developing health and strength all the time. I met one of the family in October, and was told that I would then hardly recognize these boys, so brown and rugged had they become. It is reasonable to say that this summering has added five or ten years to the lives of these children. The family above referred to are people of education and refinement, so that the influences under which the children are brought are the very best. The children become greatly attached to the family who allow them much freedom, and thus the farm-house in known as "Liberty Hall." We wish there were many such summer resorts for city boys who are suffering for the want of fresh country air, and the fresh milk and butter and bread and fruits that abound here. If the thousands of people who visit the fashionable watering places, leaving their children at home in hot, dusty cities, or bringing them with them to be pampered by rich food and fatigued by late hours, would seek for them homes for the summer at farm-houses in healthy localities, permanent benefit would result. There are plenty of such places to be found; and among the recollections of a child's life, none would be brighter or more satisfactory to look back upon than his rural experience. The highest joys and pleasures of childhood are lost in the walls of a great city.

---

BLINDERS.—One of the most unaccountable of follies is that of compelling horses to wear blinders. They are the cause of more run-aways and other accidents than all the railroads in the country. Men and horses are always more frightened at noises they cannot account for than at anything else. Allow a horse to see everything about him and he soon loses his timidity. Blinders are liable to impair the eyesight, by keeping the air from the eyes and by shortening the vision. Horses are constantly being struck by objects that they would avoid if opportunity were given them to do so. Hardly a day passes in the crowded streets of the city that we do not see horses wounded by some passing vehicle that they were unable to see until it approached too near to be avoided.

I drove a horse for two years who was never safe, and at times was almost unmanageable from fear. I took off his blinders and used an open bridle for two years more, during which time a child could drive him safely; indeed, a lad of ten years now owns him and uses him exclusively. Were he to put blinders on him again he would be unmanageable.

